# Make sex great again! - Prevalence and Treatment Options for Postcoital Dysphoria

**DOI:** 10.1192/j.eurpsy.2025.2320

**Published:** 2025-08-26

**Authors:** N. Ramalho, T. Rocha, J. F. Cunha, J. C. Moura, J. Leal, D. Seabra, I. Lopes, G. Santos, M. Rosa, A. Garcia

**Affiliations:** 1 Psychiatric and Mental Health Department, ULSAR, Barreiro, Portugal

## Abstract

**Introduction:**

Postcoital dysphoria (PCD) refers to feelings of sadness, anxiety, or irritability following sexual intercourse, even when it is consensual and satisfying. These emotions can last from minutes to hours and affect both genders.

Historically, sex was mainly viewed as a means of reproduction, with pleasure often regarded as secondary. However, during the sexual revolution of the 1960s and 70s, perceptions shifted, recognizing sex as a source of pleasure and emotional connection. Today, despite a focus on mutual satisfaction, some individuals still experience post-sexual distress, highlighting the complexity of human sexuality. As a source of important distress, PCD calls for exploration of therapeutical agents.

**Objectives:**

To examine the prevalence of postcoital dysphoria (PCD) and explore potential therapeutic agents.

**Methods:**

A non-systematic literature review using the keywords “postcoital”, “dysphoria” and “tristesse” limited to articles published in English from the PubMed®/MEDLINE® database.

**Results:**

Seven relevant studies were identified regarding postcoital dysphoria (PCD). In a study of 1,208 males, 40% reported experiencing PCT at least once, with 20% experiencing symptoms in the past month, and 3–4% regularly. PCT was linked to psychological distress, childhood sexual abuse, and sexual dysfunctions.

Among women, a UK survey of female twins found that 3.7% reported recent PCT symptoms, while 7.7% had long-term symptoms. Another study indicated that nearly 50% of female university students experienced PCT at least once, with no correlation found between PCT and relationship intimacy.

In the LGBTQIA+ population, a survey of 172 adults revealed PCD prevalence of 42% among men attracted to men and 81% among bisexual/fluid individuals. Significant correlations were observed between sex life satisfaction and PCD in this group. Regarding treatment, one single case report described a patient without psychiatric comorbidities treated successfully with escitalopram (10 mg), with symptomatic relief.

**Conclusions:**

The reviewed studies highlight the prevalence and factors contributing to postcoital dysphoria (PCD) across diverse populations. Among men, up to 40% experience PCD at least once, with contributing psychosocial factors. In women, PCT prevalence varies, with no clear link to relationship intimacy. In the LGBTQIA+ community, PCD is notably high, affecting 42% of MSM and 81% of bisexual/fluid individuals, with sexual dissatisfaction and perceived discrimination as key correlates. Treatment options remain unexplored, with only one case report showing positive results using escitalopram. These findings suggest PCD is a complex conditions influenced by psychological, sexual, and societal factors. More research is needed to understand the underlying mechanisms and explore effective treatments, particularly for minority populations and those without psychiatric comorbidities.

**Disclosure of Interest:**

None Declared

